# Interphase Engineering in Aramid-Fiber-Reinforced Epoxy Composites: From Surface Modification to Mechanical Consequences

**DOI:** 10.3390/nano16160972

**Published:** 2026-08-07

**Authors:** Minseo Kim, Yeongdam Choi, Yunsang Kim, Byounghwak Lee, Min-Kun Kim, Youngho Jin

**Affiliations:** 1 Department of Advanced Materials Engineering, Chung-Ang University, 4726 Seodong-daero, Anseong-si 17546, Republic of Korea; yy15379@cau.ac.kr (M.K.); yd7122@cau.ac.kr (Y.C.); 2 Department of Sustainable Bioproducts, Mississippi State University, 201 Locksley Way, Starkville, MS 39759, USA; ysk13@msstate.edu; 3 Department of Physics, Korea Military Academy, Seoul 01805, Republic of Korea; lebaiii@kma.ac.kr; 4 Department of Materials Science and Engineering, Myongji University, Yongin-si 17058, Republic of Korea

**Keywords:** aramid composite, interphase engineering, polydopamine, carbon nanotube, epoxy composite, hierarchical interphase

## Abstract

Aramid-fiber-reinforced epoxy composites are widely utilized in lightweight structural and protective applications; however, their performance is often limited by intrinsically weak and chemically inert fiber–matrix interfaces. Recent advances have shifted attention from conventional surface activation to deliberate interphase engineering, in which chemical functionality, hierarchical structure, and nanoscale reinforcement are integrated to regulate stress transfer and damage evolution. This review provides a comprehensive overview of interfacial design strategies for aramid/epoxy systems, including reactive surface activation, additive interphase construction, bioinspired polydopamine (PDA)-based coatings, and PDA/poly(ethyleneimine)-mediated hierarchical interphases. Particular emphasis is placed on the role of carbon nanotubes and hybrid nanofillers as interphase-active components rather than simple matrix additives, highlighting the importance of their localization, functionalization, and structural integration. This review further examines how interphase architecture influences mechanical responses under multiple loading conditions, including interlaminar shear, impact, tribological wear, viscoelastic behavior, and ballistic loading. Optimal composite performance depends on achieving an appropriate balance between strong interfacial bonding and damage-tolerant deformation mechanisms. By integrating recent experimental and conceptual developments, this review highlights the transition from “surface modification” to “interphase programming” and provides design guidelines for next-generation aramid fiber composites with improved strength, toughness, and multifunctionality.

## 1. Introduction

Aramid-fiber-reinforced epoxy composites have attracted considerable attention for lightweight structural applications owing to their high specific strength, thermal stability, abrasion resistance, chemical durability, impact tolerance, and the processing versatility associated with thermosetting matrices [1,2,3,4,5,6,7]. Nevertheless, the full mechanical potential of these composites is rarely realized because the chemically inert and relatively smooth surfaces of aramid fibers exhibit limited wettability and weak physicochemical affinity toward epoxy, resulting in insufficient stress transfer, unstable crack-arrest capability, and premature interfacial failure under multiaxial loading [1,2,3,4,5,6]. Accordingly, interphase design has emerged as a central issue in aramid/epoxy research, shifting attention from simple fiber preservation to the deliberate regulation of surface chemistry, nano-/micro-roughness, interfacial energy, and hierarchical load-transfer pathways [1,2,3,4].

Early approaches to enhance interfacial adhesion in aramid fiber composites primarily relied on chemical etching and other aggressive surface-activation techniques; however, recent studies have increasingly favored additive and architecture-building routes that improve adhesion while minimizing damage to the fiber backbone [3,4,5]. Representative strategies include amino-functional silane grafting, aramid nanofiber coating, layer-by-layer assembly, bioinspired catechol chemistry, glycidyl-polyhedral oligomeric silsesquioxanes (POSS) grafting, plasma-induced surface reconstruction, and ionic-liquid-assisted compatibilization [6,7,8,9,10,11,12,13,14,15]. These strategies increase surface polarity and create tailored interphases capable of simultaneously influencing interlaminar shear transfer, flexural resistance, dielectric behavior, thermomechanical stability, and failure evolution [4,6,7,8,9,10,11,12,13,14,15].

In this context, recent studies have demonstrated that aramid nanofiber coatings can generate chemically compatible interlayers [7]; silane-based treatments can introduce reactive groups that promote epoxy coupling [6,9]; multilayer assembly can produce controllable rough and functional films [8,11]; glycidyl-POSS can construct heat-assisted hybrid interfacial layers [12]; plasma processing can reconstruct surface aggregation states without the damage typically associated with conventional wet-chemical processes [13]; and ionic-liquid-mediated treatments can improve resin–fiber affinity via interfacial compatibilization mechanisms [14,15].

Among these approaches, polydopamine (PDA)-based modification has emerged as a versatile, substrate-independent platform that provides both adherent coatings and catechol/amine-rich reactive sites, which is particularly attractive for chemically inert aramid fibers [16,17,18]. Within these systems, PDA functions either as a standalone coating or as a foundational adhesive intermediate for secondary functionalization, such as silane grafting, metal-ion co-modification, or multilayer assembly, demonstrating robust capabilities in transforming low-activity reinforcement surfaces into chemically interactive interphases [9,10,11,19]. Nanoscale carbon fillers, including carbon nanotubes (CNTs), graphene, and hybrid nanostructures, have been extensively investigated as secondary reinforcing elements to improve the modulus, toughness, thermal transport, and damage control [20,21,22,23,24,25,26,27,28]. However, the principal challenge in realizing their full potential is not merely bulk filler addition but the effective utilization of their aspect ratio and surface chemistry through deliberate dispersion control and hierarchical interphase engineering [20,21,22,23,24,25,26,27,28]. Crucially, a stronger interface does not necessarily correspond to superior composite performance because excessively rigid interphases may suppress local yielding, frictional sliding, and other energy-dissipation mechanisms that contribute to impact tolerance and progressive failure resistance under specific loading conditions [4,10,11,14,20,21,22,23,24,25].

Therefore, the performance of aramid/epoxy composites should be interpreted through a balance between efficient stress transfer and damage-tolerant deformability rather than through interfacial shear strength alone [4,11,14,20,21,22,23,24,25]. Dynamic mechanical analysis (DMA) is particularly valuable in this regard because the storage modulus, loss modulus, and tan δ collectively reflect how the interphase design and nanofiller incorporation influence the chain mobility and viscoelastic energy dissipation [22,29]. Simultaneously, as emphasized in recent DMA-focused analyses, tan *δ* suppression should not be overinterpreted solely as a simple signature of stronger bonding because such changes may also result from variations in the rubbery modulus, constrained segmental motion, filler networking, or interphase volume fraction [29]. This broader structure–property perspective has been reinforced by recent investigations on CNT-coated aramid-fiber-reinforced epoxy composites, wherein interfacial engineering simultaneously influenced tensile behavior, hardness, thermal conductivity, and tribological response, rather than a single mechanical metric [30].

As research on aramid fiber composites has progressed, several review articles have comprehensively summarized surface modification and interfacial engineering strategies from different perspectives. As summarized in Table 1, previous reviews have emphasized additive surface modification methods, functionalization strategies, and surface/interface engineering to enhance interfacial adhesion and composite performance. The distinctive scope and perspective of the present review are summarized in Table 1.

Despite these extensive reviews and experimental studies, a unified framework linking interphase chemistry, hierarchical structure, and mechanical behavior under multiple loading conditions has not yet been comprehensively established. Therefore, a comprehensive review is required to integrate the rapidly expanding literature on aramid fiber surface modification, bioinspired interphase design, carbon-nanofiller functionalization, and viscoelastic/mechanical characterization into a unified framework [1,2,3,4,9,10,11,20,21,22,23,24,25,26,27,28,29,30]. The central premise of this review is that next-generation aramid/epoxy composites should be designed through interfacial architecture rather than isolated surface-treatment strategies. Particular attention is given to the interactions among chemical functionality, hierarchical roughness, nanoscale carbon reinforcement, and viscoelastic constraints in governing both load transfer and damage evolution [4,9,10,11,12,13,14,20,21,22,23,24,25,26,27,28,29,30]. Accordingly, this review discusses additive surface-modification strategies for aramid fibers, PDA- and poly(ethyleneimine) (PEI)-assisted hierarchical interphases, CNT/graphene-based secondary reinforcement, and the interpretation of DMA and related mechanical data to clarify how strong-yet-tolerant interfaces can be engineered for high-performance aramid/epoxy systems [1,4,9,10,11,12,16,17,18,20,21,22,23,24,25,26,27,28,29,30,33].

Table 2 summarizes the representative interphase engineering strategies reported for aramid fiber/epoxy composites, including their interphase formation modes, dominant mechanisms, mechanical improvements, and the associated tradeoffs and limitations. The comparison highlights that, although several approaches improve the interfacial strength and load-transfer capability, excessive interfacial constraints or structural complexity can introduce limitations depending on the loading condition and failure mode.

## 2. Aramid Fiber Interfacial Engineering

### 2.1. Interfacial Engineering as the Central Design Problem in Aramid/Epoxy Composites

Aramid fiber interfacial engineering primarily addresses the contradiction between the excellent intrinsic properties of aramid fibers and their limited intrinsic compatibility with thermosetting matrices. Although aramid fibers provide high specific strength, thermal resistance, and impact tolerance, their highly oriented aromatic backbone, high crystallinity, and chemically inert surface result in limited wettability and insufficient physicochemical affinity for epoxy. Consequently, the stress transfer across the fiber/matrix boundary is often incomplete, and crack initiation, interfacial debonding, and fiber pull-out may occur before the reinforcing capability of the fiber is fully utilized. Therefore, interfacial engineering extends beyond simple roughening of the fiber surface and focuses on constructing an interphase that simultaneously improves the physicochemical affinity, mechanical interlocking, and stability of stress-transfer pathways under load [1,2,3,4,5,31].

In this context, recent studies have shifted the paradigm of surface treatment from simple “activation” to deliberate “interphase design.” Earlier approaches primarily focused on introducing limited polar groups or etching the surface to improve matrix wetting. By contrast, current strategies increasingly consider the fiber surface as a platform for assembling chemically active, mechanically compliant, and occasionally multifunctional nanostructured interlayers. This shift is significant because the interfacial performance in aramid composites is governed not by a single parameter but by the coupling of surface energy, roughness, chemical reactivity, interphase thickness, and compatibility with curing chemistry [1,2,3,4,8,9,10,11,12,13,14,15,31].

### 2.2. Reactive Surface Activation: Chemical Treatment and Silane Grafting

Reactive surface activation represents one of the principal approaches in aramid-fiber interfacial engineering, whereby new functional groups are introduced onto the aramid surface to promote chemical interactions with epoxy or curing agents. A representative early example is the wet-chemical modification reported by Liu et al. [5]., in which new surface functionalities were generated to improve interfacial bonding with epoxy. Such approaches established the fundamental principle that even limited changes in surface chemistry can produce measurable improvements in adhesion when the density of reactive or polar interfacial sites is increased [5,38,39].

Building on this principle, silane grafting has emerged as one of the most practical and widely adopted strategies because silane molecules can form molecular bridges between the fiber surface and organic epoxy network. In particular, amino-functional silane grafting significantly increases the surface roughness and reactive group density, thereby strengthening the interfacial interaction and improving the load-transfer efficiency of aramid-fiber-reinforced composites. More recent studies on secondary silane grafting have further suggested that sequential or multistep silanization can enhance this effect by producing more stable and better-distributed interfacial chemistry than conventional one-step treatments. Such improvements are particularly valuable for textile-form aramid reinforcements where surface accessibility and treatment uniformity are critical [6,33].

From a design perspective, these chemically reactive routes are attractive owing to their conceptual simplicity and compatibility with conventional composite processing. However, a chemical increase in adhesion does not necessarily produce a hierarchically optimized interphase. If the treatment is excessively aggressive, the fiber integrity may be compromised, whereas if it is extremely mild, the gain in interfacial performance may remain limited. Accordingly, recent studies have increasingly combined chemical activation with additive coating or hybrid interlayer construction to simultaneously achieve chemical bonding and microstructural roughening [5,6,33].

### 2.3. Additive and Architecture-Building Strategies: From Nanofiber Coatings to Layer-by-Layer Interphases

The second and increasingly influential route involves additive interfacial engineering [32,40], in which a new interphase is intentionally constructed on the aramid surface instead of relying only on direct surface activation. This approach is particularly attractive for aramid fibers because it minimizes backbone damage while enabling finer control over the interphase composition and morphology [41]. For example, aramid nanofiber (ANF) coatings provide an interesting strategy because they create a chemically homologous interlayer between the parent aramid fiber and the surrounding polymer matrix. Such coatings can increase the surface roughness, effective contact area, and stress transfer while preserving the intrinsic characteristics of the reinforcement [7,42].

Layer-by-layer self-assembly extends this concept by enabling the fabrication of controllable multilayer films on aramid surfaces. Compared with single-step activation, layer-by-layer assembly offers a more modular route for balancing roughness, charge, interfacial energy, and functional group density. The resulting interphases are often more uniform and tunable and can be designed to cooperate with epoxy curing reactions while also enhancing mechanical interlocking. The broader significance of this strategy is that it transforms the interface into a programmable region rather than a passive boundary, enabling researchers to tune the interfacial architecture according to target properties, such as the interlaminar shear strength (ILSS), flexural performance, or dielectric response [8,43].

This architecture-building perspective is reinforced by a recent glycidyl-POSS-assisted interfacial design. In this study, heat-assisted treatment and POSS grafting were used to construct a hybrid interfacial layer that exhibits enhanced chemical compatibility with epoxy and greater structural resistance to interfacial failure. This result shows that interfacial engineering can benefit from cage-like molecular building blocks that simultaneously contribute to nanoscale rigidity, reactive functionality, and controlled interphase thickness. Collectively, aramid nanofiber coating, layer-by-layer assembly, and POSS-assisted grafting demonstrate that the future of aramid interfacial engineering lies in the construction of tailored interphases rather than simply altering the native surface [7,8,12,44].

### 2.4. Bioinspired Interphases: Polydopamine as a Universal Adhesive Platform

Among the various interfacial strategies proposed for aramid fibers, PDA-based modification has emerged as one of the most versatile strategies because it provides a substrate-independent route for surface functionalization under relatively mild conditions. Inspired by mussel adhesion chemistry, dopamine can be used to form adherent coatings on a broad range of materials via oxidative self-polymerization while simultaneously introducing catechol and amine functionalities. In aramid systems, this indicates that a surface previously considered chemically inert can be transformed into a reactive interfacial platform capable of anchoring silanes, polymers, or nanocarbon fillers [9,16,17,18].

The importance of PDA in aramid composites is not limited to improving wettability. Rather, PDA functions as an interfacial bridge layer that can couple the surface chemistry, nano-/microscale roughness, and secondary functionalization. This role was demonstrated in the bioinspired PDA-plus-epoxy-functionalized silane system, in which the PDA layer enabled more effective grafting and substantially enhanced adhesion. Similarly, ZnCl_2_/PDA co-modification demonstrated synergistic interactions between PDA and other treatments to improve the mechanical performance of the composites. These studies suggest that PDA is particularly valuable when the design objective is to move beyond simple adhesion enhancement toward robust and multifunctional interphase construction [9,10].

**Figure 1:**
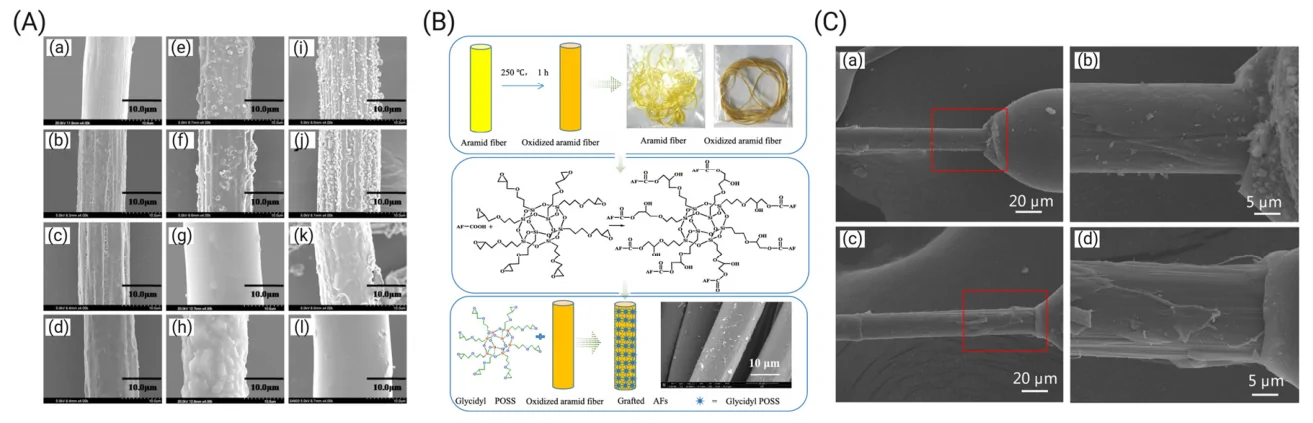
Representative scanning electron microscopy (SEM) images and schematics of aramid fiber surface modification through different interphase-engineering strategies: (**A**) PDA-based surface modification: SEM images of (**a**) untreated meta-aramid (MPIA) fibers, (**b**) PDA-coated MPIA fibers, (**c**–**h**) PDA/KH560-modified fibers prepared under different modification conditions, and (**i**–**l**) PDA/KH560-modified fibers prepared with different KH560 concentrations. (**B**) Glycidyl POSS grafting: heat-oxidation pretreatment of aramid fibers (**top**), the grafting reaction between glycidyl POSS and oxidized aramid fibers (**middle**), and the formation and surface morphology of glycidyl POSS-grafted aramid fibers (**bottom**). (**C**) Silane-based surface functionalization: (**a**) an untreated aramid fiber/epoxy microcomposite after fiber pull-out, (**b**) an enlarged view of the red-boxed region in (**a**), (**c**) an APS-grafted aramid fiber/epoxy microcomposite after fiber pull-out, and (**d**) an enlarged view of the red-boxed region in (**c**). The APS-grafted fiber retains more epoxy fragments on its surface. These approaches generate distinct interphase morphologies and surface chemistries that influence fiber–matrix interactions in aramid-fiber-reinforced composites [6,9,12]. Images reproduced with permission from Ref. [6]. Copyright Wiley, and Refs. [9,12]. Copyright American Chemical Society.

This foundational role of PDA enables the development of even more advanced hierarchical interphases. Rather than acting as a simple monolayer coating, PDA acts as the primary anchoring platform for subsequent polymer or nanomaterial assemblies. Consequently, the interphase evolves into a gradient mechanical zone rather than a sharp boundary. By providing a highly reactive base, PDA enables subsequent construction of multiscale load-transfer pathways. Evidence from both advanced aramid and broader carbon fiber studies further supports this interpretation, demonstrating that PDA serves as an effective platform for converting low-activity reinforcement surfaces into highly interactive and structurally integrated composite interfaces [11,19]. Representative PDA-based interphases, together with POSS-assisted and silane-based interphase-engineering strategies, are illustrated in Figure 1.

### 2.5. Emerging Scalable Routes: Plasma Reconstruction and Ionic-Liquid-Assisted Compatibilization

For practical composite manufacturing, interfacial engineering methods must be evaluated not only by laboratory-scale performance gains but also by their scalability, processing compatibility, and potential damage to the fiber. Plasma treatment has attracted considerable attention because it can alter the surface chemistry and morphology without requiring large quantities of solvents or prolonged chemical reaction steps. Recent plasma-based studies on aramid fibers have gone beyond conventional surface activation and proposed the concept of reconstructing the surface aggregation state, indicating that plasma can reorganize the outermost structural features of the fiber to improve subsequent interfacial interactions with the matrix. This development represents an important conceptual shift because it frames plasma treatment as a structural engineering tool, rather than merely a cleaning or oxidation method [13,35].

Ionic-liquid-assisted compatibilization represents another promising approach. Imidazolium-based ionic liquids enhance aramid–epoxy interactions and improve the performance of aramid-containing epoxy composites. Their significance lies in their ability to modify the interfacial affinity while potentially assisting in resin wetting and local molecular mobility at the interface [45]. Compared with strongly oxidative or aggressive chemical treatments, ionic-liquid-based approaches may offer a milder yet more effective means of improving fiber/matrix compatibility. Moreover, when considering PDA-, silane-, and plasma-based methods [46], ionic liquids broaden the design space by demonstrating that the interphase can also be tuned using molecular-level compatibilizers rather than solely through covalent grafting or deposited solid layers [14,15].

These emerging methods highlight a broader trend in aramid interfacial engineering: the field is moving toward strategies that are simultaneously more controllable, multifunctional, and manufacturing-aware. Plasma reconstruction, ionic-liquid compatibilization, and hybrid interlayers, such as glycidyl-POSS, all provide the same conclusion: high-performance aramid/epoxy composites require an interphase that is chemically interactive, mechanically stable, and process-compatible across multiple length scales [12,13,14,15,35].

### 2.6. Section Summary and Design Implications

**Figure 2:**
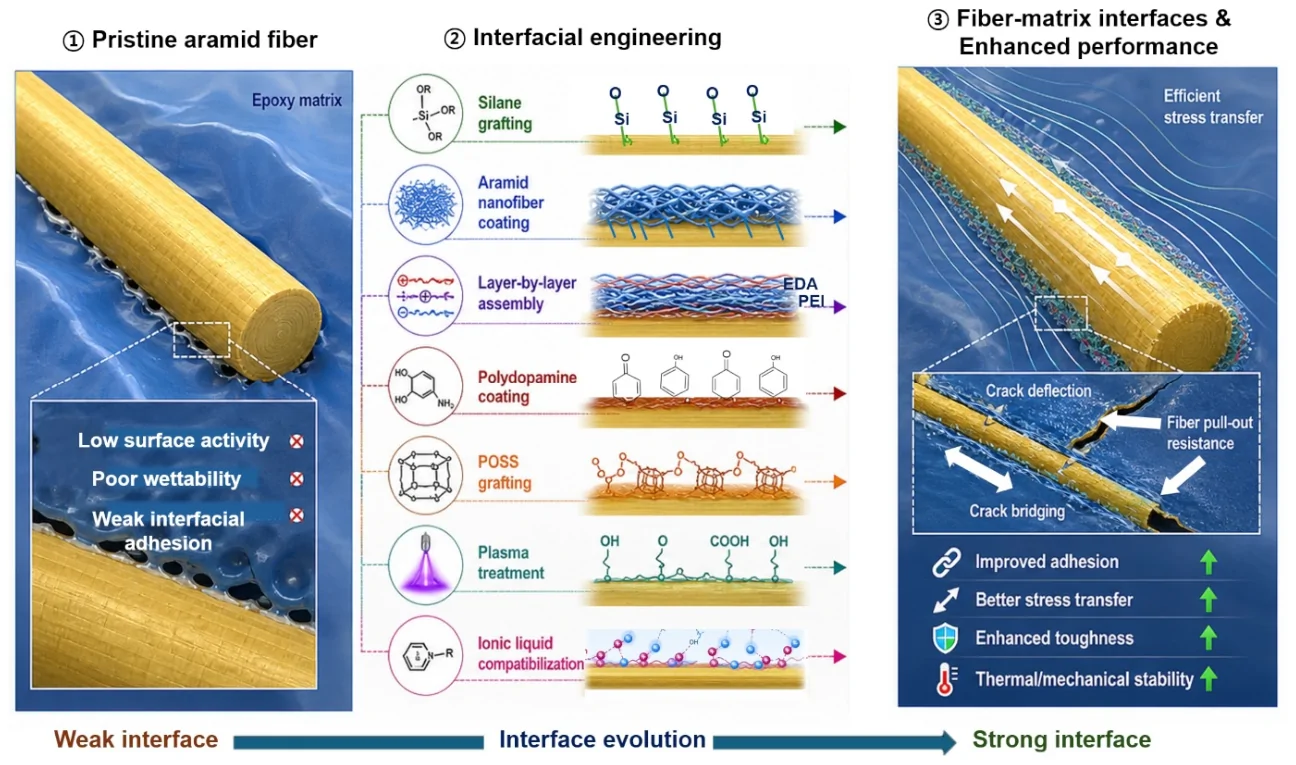
Conceptual schematic summarizing representative interfacial engineering approaches for aramid fiber/epoxy composites, including silane grafting, nanofiber coating, layer-by-layer assembly, PDA coating, POSS grafting, plasma treatment, and ionic-liquid compatibilization, based on previous literature reports [6,7,8,9,10,11,12,13,14,15,33,35,42,43,44,45,46].

Overall, recent studies have suggested that aramid fiber interfacial engineering has evolved from simple surface activation to the deliberate construction of chemically and mechanically integrated interphases. Current strategies increasingly combine reactive surface functionalization, additive interphase construction, and hierarchical nanostructuring to simultaneously improve interfacial adhesion, stress transfer, and damage tolerance [6,7,8,9,10,11,12,13,14,15,33]. In this context, PDA-assisted multilayers, nanofiber coatings, plasma-assisted activation, and POSS- or ionic-liquid-based hybrid interphases have attracted increasing attention because they provide more tunable and multifunctional interfacial architectures than single-step chemical treatments. However, maximizing interfacial bonding does not always guarantee the optimum composite performance because excessively rigid or highly constrained interphases may reduce the deformability, processability, and energy-dissipation capability, depending on the loading condition and failure mode [1,2,3,4,11,12,13,14,15,31]. Figure 2 summarizes the evolution of aramid fiber interfacial engineering from chemically inert fiber surfaces to structurally integrated and multifunctional interphase architectures.

## 3. PDA/PEI-Based Interphase Design

### 3.1. Rationale for PDA/PEI-Based Interphase Design

Although a standalone PDA coating (as discussed in Section 2.4) successfully activated the inert aramid surface, the co-deposition or sequential assembly of PDA with PEI elevated the interface from a simple primer to a multifunctional, multilayered architecture. This design concept originates from the complementary roles of the two components: PDA ensures universal and robust adhesion to aramid fibers via catechol groups, whereas PEI introduces a high density of amine functionalities. This high amine concentration is crucial because it fundamentally changes the reactivity of the layer, enabling it to act as a powerful structural linker for secondary components, most notably CNTs and other nanofillers. Consequently, PDA/PEI is not only a surface modifier but also a dynamic platform for hierarchical interphase construction [9,16,17,18,19].

This multilayering capability is particularly transformative for aramid fibers. A PDA-only layer improves wetting; however, the PDA/PEI network creates a structurally adaptable, three-dimensional interphase capable of deep chemical integration. PEI serves as an essential “molecular glue” or “linker” that electrostatically and covalently binds to the functionalized nanocarbon species, assembling them into a dense interfacial zone. This transition from a passive two-dimensional coating to an active multiscale nanocomposite interphase is highly relevant for high-performance composites, where the interface must not only adhere to the matrix but also actively mediate stress transfer, crack deflection, and local energy dissipation under complex loading conditions [11,19,47,48,49].

**Figure 3:**
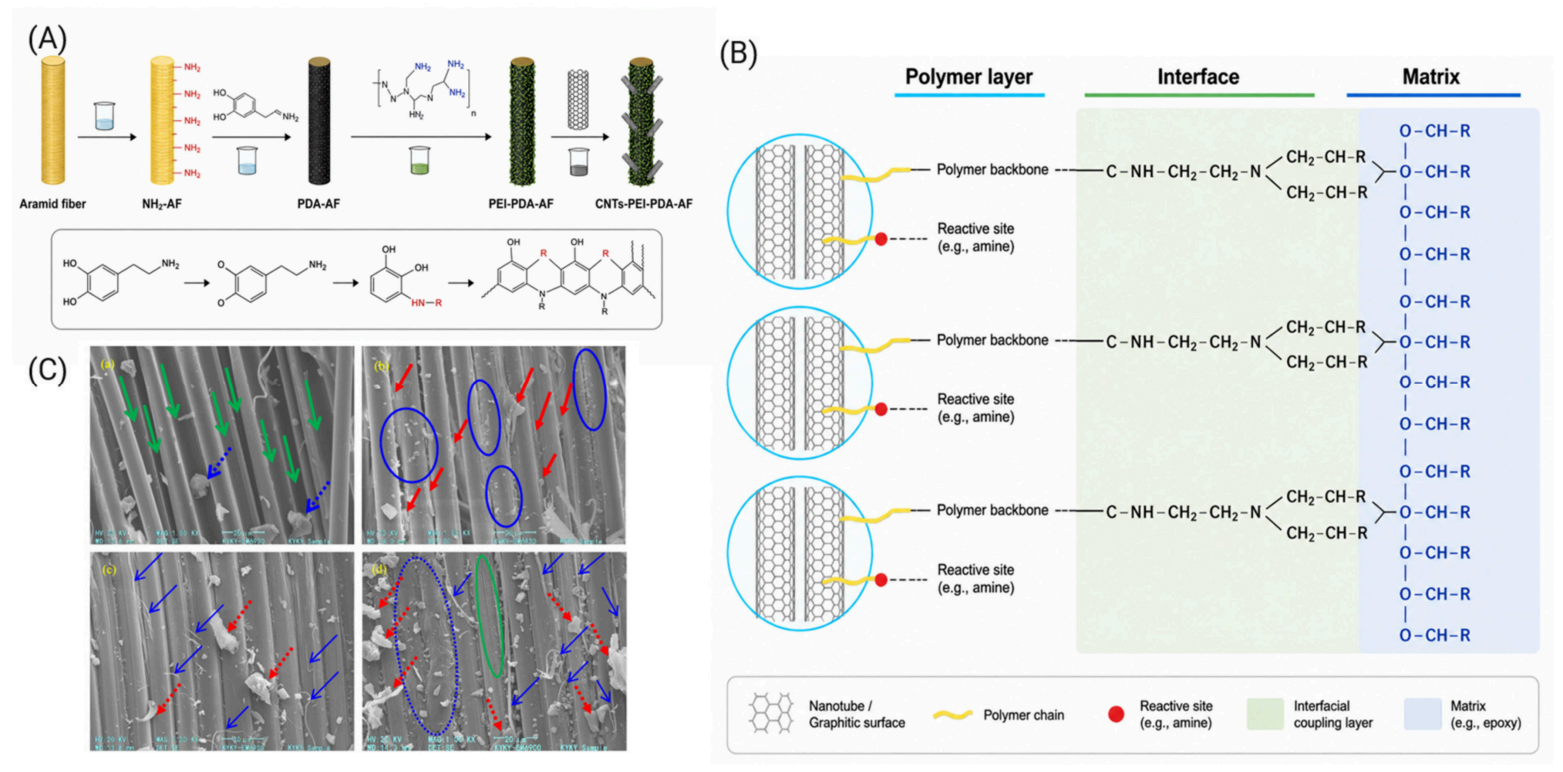
Stepwise formation and interfacial structure of NH_2_-CNTs-PEI-PDA multilayer-modified aramid fiber/epoxy composites. (**A**) Surface modification process involving sequential PDA coating, PEI assembly, and NH_2_-CNT immobilization, together with the corresponding reaction mechanism. (**B**) Schematic of interfacial coupling between the NH_2_-CNTs-PEI-PDA multilayer and the epoxy matrix. (**C**) SEM images of tensile fracture surfaces of (**a**) R-AF/EP, (**b**) PDA-AF/EP, (**c**) PEI-PDA-AF/EP, and (**d**) NH_2_-CNTs-PEI-PDA-AF/EP, showing the transition from interfacial debonding to a more integrated fiber–matrix interphase. In (**a**), green and blue dotted arrows indicate fiber pull-out channels and brittle epoxy fragments, respectively; in (**b**), blue circles and red arrows indicate fragmented and adherent epoxy; in (**c**), red dotted and blue arrows indicate plastically deformed epoxy debris and broken fiber filaments; and in (**d**), red dotted arrows, blue dotted circles, green circles, and blue arrows indicate deformed epoxy debris, dense matrix coverage, shear-plasticization regions, and thick interphase structures, respectively. Reproduced from Ref. [11]. Copyright ACS Omega.

Figure 3 shows that PDA/PEI/NH_2_-CNT deposition converts the inert aramid surface into a more reactive and roughened interphase, which improves coupling with the epoxy matrix. The corresponding fracture morphologies indicate reduced interfacial debonding and more integrated fiber–matrix failure after multilayer modification, supporting the formation of a stronger load-bearing interphase [11].

### 3.2. Deposition Chemistry and Interphase Formation Mechanism

The effectiveness of the PDA/PEI-based interphases strongly depends on the formation and organization of the coating on the fiber surface. PDA originates from the oxidative self-polymerization of dopamine, and its broad substrate compatibility makes it a key bioinspired surface modification tool for advanced composites [16,17,18]. However, when PEI is incorporated during or after the deposition, the interphase can no longer be viewed as a simple PDA film. Instead, the resulting layer is better understood as a catechol/polyamine-rich interfacial network in which the adhesion, amine functionality, and secondary assembly behaviors are coupled.

This distinction is important because the PDA/PEI layer governs substantially more than surface chemistry. Co-deposition studies have shown that the formation kinetics and morphology of PDA/PEI coatings depend sensitively on the solution composition, deposition conditions, and substrate characteristics, indicating that the interphase thickness, roughness, continuity, and chemical density are controllable parameters rather than fixed outcomes [49]. From an interfacial engineering perspective, this behavior indicates that the PDA/PEI platform can be tuned to deliver either relatively thin reactive primer layers or more developed interphases capable of anchoring additional nanoscale building blocks. Mechanistic studies on dopamine deposition and related fiber systems have further suggested that the local arrangement of catechol- and amine-containing species strongly influences the final adhesion state and interaction of the modified surface with epoxy matrices [50].

Accordingly, the key advantage of the PDA/PEI interphase design is its modularity. PDA establishes strong and versatile interfacial anchoring, whereas PEI enriches the deposited layer with accessible amine groups and enhances electrostatic and hydrogen-bonding interactions. Collectively, these features create an interphase that is more suitable for subsequent functionalization than an untreated aramid surface or a single-component coating. Therefore, PDA/PEI systems have been increasingly employed not only as direct adhesion-promoting layers but also as interfacial platforms for multilayer growth, nanofiller immobilization, and hybrid interphase construction [11,34,47,48,49,50].

### 3.3. PDA/PEI-Based Hierarchical Interphase Engineering: Mechanisms, Nanocarbon Integration, and Design Considerations

Since the fundamental deposition mechanism and adhesion characteristics of PDA have already been discussed in Section 2.4, the present section focuses on the additional functionalities introduced by polyethyleneimine (PEI) and their implications for programmable interphase engineering. Rather than functioning solely as an adhesion-promoting coating, the PDA/PEI system serves as a chemically active bridge interphase that enables subsequent functionalization and hierarchical interface construction. The amine-rich PEI layer expands the chemical versatility of PDA by providing abundant reactive sites for the immobilization of nanofillers and improving the compatibility between the chemically inert aramid fiber surface and the epoxy matrix [9,11,47]. The effectiveness of this strategy is well demonstrated in multilayer PDA/PEI/nanocarbon systems. In representative PDA/PEI/NH_2_-CNT interphases, amino-functionalized CNTs are immobilized within the amine-rich interphase, producing additional load-transfer pathways, increasing nanoscale roughness, and improving interfacial continuity. Consequently, wettability, interfacial energy, and stress-transfer efficiency are enhanced simultaneously [11]. Similar hierarchical architectures have also been developed using graphene oxide, where the PDA/PEI layer acts as an enabling platform for the sequential assembly of GO nanosheets, illustrating that its primary role is to provide a versatile framework for hierarchical interphase construction rather than simply improving surface adhesion [47]. Related studies on carbon-fiber composites further demonstrate that the cooperative interaction between catechol and polyamine chemistry represents a broadly applicable interphase design strategy rather than a mechanism unique to aramid fibers [48].

From an interphase-engineering perspective, the greatest advantage of PDA/PEI systems lies in their ability to integrate reactive functional groups, nanofillers, and mechanically relevant interface architectures within a single boundary region. Compared with conventional surface activation methods that mainly increase polarity or surface roughness, PDA/PEI-based interphases provide a more programmable and multifunctional platform capable of simultaneously improving wetting, interfacial continuity, and multiscale stress transfer [11,34,47,48,49,50]. Moreover, the mild deposition conditions are particularly attractive for aramid fibers because they minimize damage to the intrinsic fiber structure while allowing versatile secondary functionalization with CNTs, GO, and other nanomaterials.

Nevertheless, several limitations and design tradeoffs remain. The morphology and thickness of PDA/PEI coatings are strongly influenced by deposition parameters such as pH, concentration, deposition time, and substrate characteristics, making reproducible interphase construction challenging [49]. Furthermore, maximizing interfacial adhesion does not necessarily result in optimal composite performance. Excessively thick or highly constrained interphases may introduce local stress concentrations, restrict resin penetration, reduce interfacial deformability, and suppress energy-dissipation mechanisms under complex loading conditions [11,34,49,50]. Therefore, future PDA/PEI interphase design should emphasize balancing chemical bonding, interphase compliance, nanofiller organization, and processing reproducibility rather than maximizing individual interfacial properties alone. More importantly, quantitative structure–property relationships linking coating chemistry, interphase morphology, cure behavior, and local failure mechanisms remain relatively limited in aramid/epoxy systems and should become a major focus of future research toward predictive and multifunctional interphase engineering [34,48,49,50].

### 3.4. Practical Considerations for Large-Scale Surface Modification

Although considerable progress has been made in improving the interfacial performance of aramid fibers, practical manufacturing considerations are equally important for their industrial implementation. Conventional wet-chemical approaches, including dopamine/polyphenol coating and chemical grafting, provide versatile surface functionalization but generally require prolonged reaction times, solvent-intensive processing, and repeated washing and drying steps. These characteristics increase processing complexity and reduce compatibility with continuous textile manufacturing. In contrast, plasma treatment offers rapid, solvent-free surface activation and can be readily integrated into roll-to-roll processing, although excessive plasma exposure may induce surface damage and requires specialized equipment. Supercritical CO_2_-assisted modification has recently attracted increasing attention because it combines low solvent consumption with high diffusivity, enabling homogeneous functionalization while largely preserving the intrinsic properties of aramid fibers. However, the requirement for high-pressure processing systems remains a practical limitation for large-scale adoption [3,32,51,52,53].

### 3.5. Systematic Comparison for Industrial Engineering Applications

While the chemical and mechanical enhancements of individual interphase engineering strategies have been detailed in the preceding sections, their industrial implementation requires a rigorous evaluation of manufacturing efficiency and economic feasibility. For engineering-grade composites, the transition from laboratory-scale to industrial production is governed by the “manufacturability-performance” trade-off. Table 3 provides a systematic comparison of the most prominent modification methods across four critical engineering metrics: solvent consumption, processing cycle time, intrinsic fiber strength loss, and scale-up.

As summarized in Table 3, atmospheric pressure plasma treatment remains as promising candidate for high-speed continuous production due to its dry, solvent-free nature and compatibility with on-line processing [51]. In contrast, additive methods such as ANF and PDA coatings, while superior in maintaining fiber integrity and “repairing” surface defects [56], are currently hindered by slow kinetics and significant chemical handling requirements, making them more suitable for high-value-added batch production [1]. Supercritical CO_2_ modification offers unique advantages for uniform treatment of complex woven structures without solvent waste, yet the high capital expenditure for high-pressure systems remains a barrier to broader adoption [57]. Therefore, future industrial interphase engineering should optimize not only interfacial performance but also manufacturing cost, energy consumption, solvent footprint, and compatibility with continuous composite production.

## 4. CNT and Hybrid Nanofiller Effects

### 4.1. CNTs as Interphase-Active Nanofillers Rather than Simple Matrix Additives

CNTs have attracted sustained attention in fiber-reinforced thermosets because their high aspect ratio, nanoscale diameter, and multifunctional surface chemistry allow them to operate as highly effective interphase modifiers and not merely as bulk fillers. In epoxy systems, CNTs can simultaneously promote crack deflection, local plastic deformation, pullout energy dissipation, and improved stress transfer provided that the dispersion and interfacial compatibility are properly controlled. From a review perspective, the key conceptual shift is that CNTs should not be regarded only as stiffness-enhancing additives in the resin-rich region but also as interphase-active building blocks whose greatest value emerges when they are localized at or near the fiber/matrix boundary [23,58,59,60].

### 4.2. Functionalization and Dispersion for Determining Whether CNTs Toughen or Merely Aggregate

The early CNT/epoxy literature established a principle that remains central today: the benefits of CNT addition are highly nonlinear and strongly dependent on the dispersion quality and nanotube surface state. High loading leads to microscale agglomerates that serve as severe stress raisers, transitioning the fracture mode from ductile matrix yielding to brittle crack propagation [61]. Even very low CNT contents can increase the stiffness and fracture toughness when the dispersion is sufficiently uniform. Earlier studies on surface-modified CNTs highlighted the importance of interfacial tailoring for effective reinforcement [22,58,62]. Later studies further clarified that noncovalent or mild functionalization strategies can improve the dispersion and interfacial interactions without excessively damaging the intrinsic nanotube structure. For example, melamine-functionalized CNTs yield marked gains in the modulus, strength, and toughness of epoxy, illustrating that interfacial engineering is as important as nanotube loading [23]. Accordingly, in reviewing CNT-reinforced aramid/epoxy systems, the decisive question is not whether CNTs are present, but whether they are chemically compatible, spatially distributed, and structurally well-preserved enough to participate in load-transfer and crack-arrest mechanisms [23,58,59,62].

### 4.3. Increased Effectiveness of Interphase-Localized CNTs Compared with Bulk-Dispersed CNTs in Structural Composites

A major advancement in this field is the relocation of CNTs from the bulk epoxy phase to the fiber/interphase region. This strategy is particularly attractive for structural composites because it precisely concentrates on nanoscale reinforcement, where stress transfer, debonding, and crack initiation are most critical. Comparative studies on CNT- and GO-coated carbon fibers demonstrated that CNT-coated fibers produced a markedly higher interfacial shear strength, whereas GO offered certain advantages in environmental resistance, highlighting how nanofiller geometry and chemistry differentially shape interphase performance [26]. Similarly, through-thickness vertically aligned CNT architectures have been shown to enhance the interlaminar shear response and shift failure from adhesive to more cohesive modes, confirming that CNTs can function as nanoscale bridges across weak interfaces [25]. Although some of these studies were conducted on carbon-fiber systems, the interphase-level design logic directly informs aramid/epoxy composites, where poor surface activity limits efficient stress transfer. This relevance is reinforced by recent aramid-based reports showing that short CNTs can substantially improve interfacial bonding and overall mechanical performance and that CNT-coated aramid fiber/epoxy composites can also improve tribological and structural responses [27,30].

### 4.4. Extension of CNT Reinforcement from Single-Function Toughening to Hierarchical Interphase Design by Hybrid Nanofillers

The next stage of development was the emergence of hybrid nanofiller concepts, in which CNTs are combined with complementary nanomaterials or coupling layers to create hierarchical interphases. In such systems, one-dimensional CNTs provide crack-bridging ability, nanoscale roughness, and conductive pathways, whereas two-dimensional components, such as GO, contribute to broader surface coverage, barrier effects, and additional reactive sites. When integrated with adhesive interlayers, such as PDA/PEI-derived coatings, CNTs can be immobilized more uniformly and transformed from loosely dispersed fillers to organized interphase constituents [34,47]. This hybridization strategy is conceptually important because it moves the design target away from maximizing a single property and balances stiffness, toughness, wetting, interfacial bonding, and processing stability. The literature also clarifies that hybrid systems are not automatically superior. If the added nanophases increase the viscosity, encourage agglomeration, or create an overly thick and brittle interphase, the apparent gains in one metric may be offset by losses in another [26,28,59,60].

### 4.5. Other Nanofillers Beyond CNTs and GO: Oxide, Clay, Ceramic, and Emerging 2D/Porous Nanophases

#### 4.5.1. Nanoclay and Layered Silicates

In addition to CNTs and GO, nanoclay-based fillers provide a relatively simple and scalable route for improving the aramid/epoxy interphase. In Kevlar/epoxy systems, organo-modified bentonite has been shown to improve tensile, flexural, and shear-related properties when the filler is well dispersed and used at moderate loading. The main benefit of nanoclays is not merely matrix stiffening but also the creation of a more tortuous crack path and a more constrained local resin region around the fiber, which can delay debonding and improve stress transfer. However, these benefits remain strongly loading-dependent [63].

#### 4.5.2. Oxide Nanoparticles: SiO_2_, TiO_2_, ZnO, and ZrO_2_

Among noncarbon nanofillers, oxide nanoparticles provide some of the clearest evidence for interphase improvement in aramid-based epoxy composites. Surface-grown nano-SiO_2_ on aramid fibers has been reported to markedly increase the interfacial shear strength while also improving the UV resistance and thermal stability, indicating that silica can act simultaneously as a roughening phase and a multifunctional protective layer [54]. Similarly, TiO_2_-coated aramid fibers showed substantial gains in interfacial shear strength in conjunction with improved UV durability, whereas ZnO nanoparticle sizing increased the interfacial strength and helped retain the mechanical properties after UV exposure [64,65]. In addition, oxide nanoparticle toughening at the laminate level, including SiO_2_- and ZrO_2_-modified aramid/epoxy-based systems, has been associated with an improved impact response. Collectively, these studies suggest that oxide nanoparticles are effective when immobilized at the fiber surface or interphase, where they can simultaneously increase surface activity, mechanical interlocking, crack pinning, and environmental stability [66].

#### 4.5.3. Ceramic Nanoparticles and Mixed Inorganic Coatings

Ceramic nanoparticles, such as SiC and Al_2_O_3_, have been investigated less extensively than CNTs, GO, or oxide nanoparticle coatings in aramid/epoxy systems; however, the available results indicate promising interfacial potential. Comparative coating studies on Kevlar fiber mats have shown that SiC, Al_2_O_3_, and graphene nanoparticle surface deposition can improve surface roughness, thermal resistance, and mechanical response, supporting the idea that ceramic nanoparticles may function as effective secondary interphase reinforcements when properly anchored to the fiber surface [67]. However, the literature on SiC- or Al_2_O_3_-specific aramid/epoxy interphase designs remains limited [68]. Therefore, these fillers are presented as emerging or exploratory candidates, particularly for applications in which wear resistance, thermal durability, and impact tolerance are as important as static strength.

#### 4.5.4. Emerging Nanofillers: MXene and MOF-Based Concepts

Recent studies have also indicated a broader transition from conventional nanoparticle toughening to a multifunctional interphase design using emerging nanofillers. MXenes are particularly attractive because of their two-dimensional morphology, hydrophilic surface terminations, and high surface activity, which favor localization at the fiber–resin boundary rather than simple bulk dispersion in epoxy. Specifically, the intrinsic surface chemistry of Ti_3_C_2_T_x_ MXene, characterized by Ti-terminated functional groups, facilitates robust interfacial interactions with the epoxy matrix’s oxygen atoms even without extensive pretreatment. This chemical compatibility is crucial for establishing long-term stability at the aramid-epoxy boundary [69]. Recent reviews of MXene/epoxy systems have noted that these materials are introduced predominantly at the fiber–resin interface, and this logic is directly relevant to aramid/epoxy composites, where interfacial inactivity remains a major limitation [70]. Metal–organic framework (MOF)-based nanofillers are at an earlier stage in this field; however, their porous and chemically tailorable structures make them interesting candidates for future hierarchical interphases that combine adhesion promotion with barrier, sensing, or flame-retardant functions [71]. Furthermore, the evolution toward ‘MOF-on-MXene’ hierarchical architectures demonstrates a synergistic strategy that overcomes MXene agglomeration while providing a ‘rigid-internal, flexible-external’ interphase designed for dynamic stress dissipation [72]. Accordingly, MXene- and MOF-based approaches are best framed not as established replacements for CNT/GO but as next-generation platforms for multifunctional interphase engineering.

### 4.6. Section Summary and Implications for Aramid/Epoxy Composites

Overall, the literature indicates that the performance of CNT-reinforced composites is governed less by the presence of CNTs than by their precise placement, chemistry, or structural integration. For aramid/epoxy systems, CNT reinforcement is most persuasive when discussed as an extension of the interphase engineering strategies outlined in Section 2 and Section 3. As aramid fibers possess a chemically inert surface, simply adding CNTs to a bulk epoxy matrix is unlikely to fully exploit their reinforcing capabilities.

Instead, three consistent design lessons have emerged: localized interphase reinforcement outperforms indiscriminate bulk loading; functionalization must balance bonding with nanotube integrity; and hybrid strategies (such as PDA/PEI-assisted assembly) offer the most robust improvements. When CNTs are introduced through these surface-mediated routes, they are transformed from isolated nanoadditives into organized interphase constituents. This hierarchical integration simultaneously improves wettability, creates mechanical interlocking sites, and constructs multiscale pathways for load redistribution [39,42]. Consequently, such interphases may contribute to improved tensile and flexural behaviors while also influencing wear resistance, frictional stability, and damage tolerance, depending on the interphase structure and loading conditions [27,30].

These findings strongly support a conceptual transition from “CNT-filled epoxy” toward “CNT-programmed interphases,” where carbon nanofillers operate synergistically with surface chemistries to produce a stronger, tougher, and multifunctional interface [23,26,27,28,30,34,47,48,49,50,58,59,60,62].

**Figure 4:**
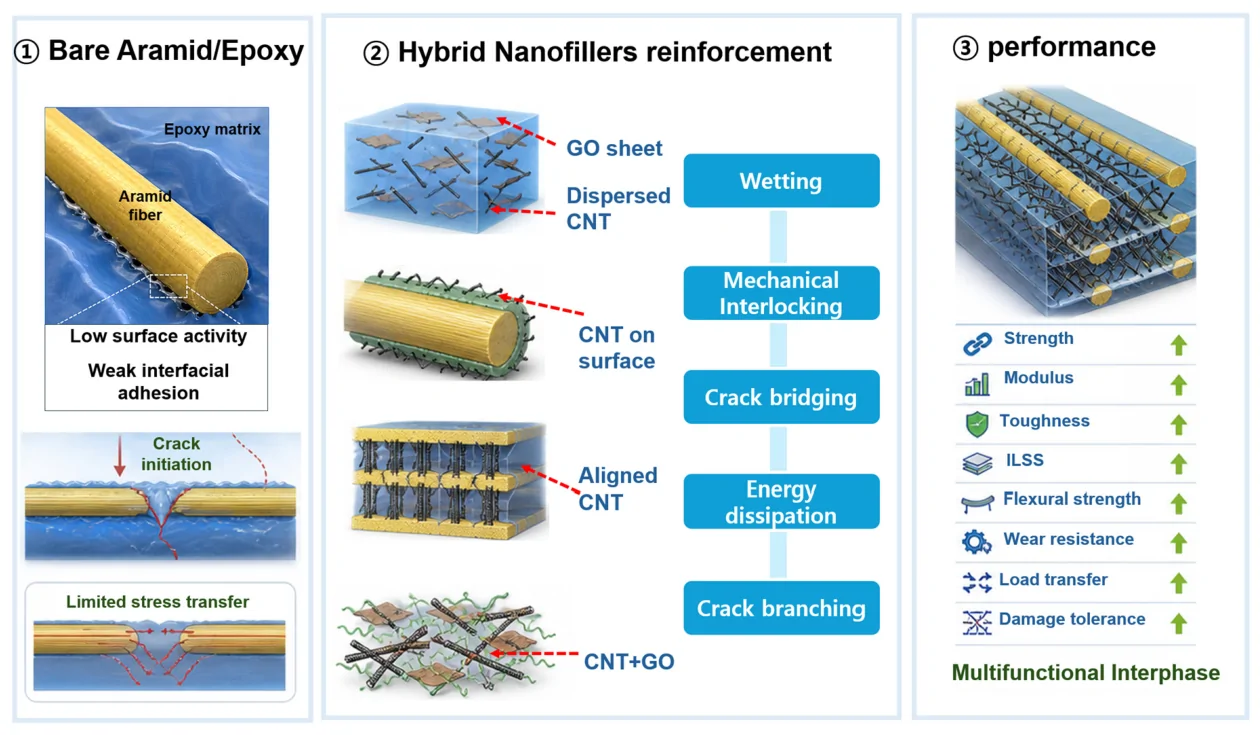
CNT- and hybrid-nanofiller-enabled interphase engineering in aramid fiber/epoxy composites, highlighting structure–mechanism–performance relationships from weak interfaces to multifunctional, load-bearing architectures.

Figure 4 summarizes how CNTs and hybrid nanofillers transform the aramid fiber/epoxy interphase from a weak, poorly wetted interface into a rougher, more reactive, and load-bearing architecture. Through improved wetting, mechanical interlocking, crack bridging, pullout, and interlaminar reinforcement, these nanoscale modifications can improve interfacial load transfer, crack deflection behavior, and select mechanical or multifunctional responses.

## 5. Nanofiller-Enabled Mechanical Consequences of Interphase Engineering

**Figure 5:**
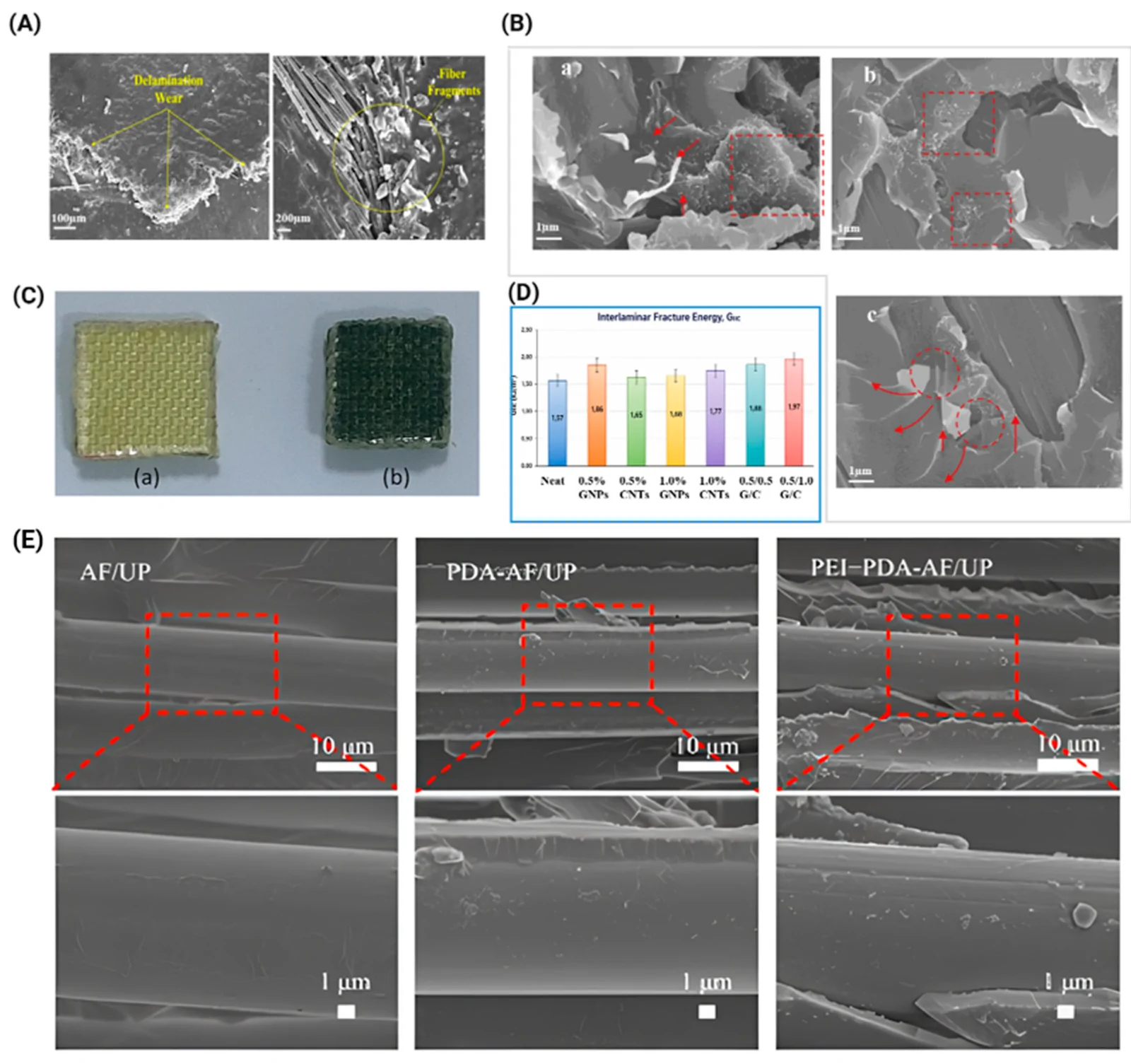
Representative fracture morphologies and interfacial failure mechanisms reported for fiber/epoxy composites with engineered interphases: (**A**) SEM images of worn surfaces showing delamination wear (**left**) and fiber fragmentation (**right**) [30]. (**B**) SEM images of hybrid failure mechanisms in a multi-walled carbon nanotube (MWCNT)/graphene nanoplatelet (GNP) composite: (**a**) coexistence of MWCNTs and GNPs within the matrix; (**b**) MWCNTs attached to GNPs; and (**c**) crack bifurcation and pull-out behavior of MWCNTs and GNPs. Red arrows indicate crack branching and pull-out mechanisms, while red dashed boxes and circles highlight MWCNT–GNP interaction regions and GNPs responsible for crack bifurcation, respectively [24]. (**C**) Optical images of (**a**) aramid fiber/epoxy and (**b**) CAF-FCNT/epoxy composites [30]. (**D**) Mode II interlaminar fracture energy of the composites [24]. (**E**) Representative SEM images of tensile fracture surfaces of AF/UP (unsaturated polyester), PDA-AF/UP, and PEI–PDA-AF/UP composites: AF/UP with smooth fiber surfaces and little residual UP matrix (left); PDA-AF/UP with increased residual matrix adhesion on the fiber surfaces (middle); and PEI–PDA-AF/UP with pronounced matrix residues and multilayer peeling features (right) [74]. Panels (**A**–**C**) were reproduced from Refs. [24,30] with permission from Elsevier. Panel (**D**) was redrawn based on data from Ref. [24]. Panel (**E**) was reproduced from Ref. [74].

The mechanical consequences of interphase engineering in aramid fiber/epoxy composites are manifested under various loading conditions, including impact, shear, sliding, and high-rate deformation. As discussed in Section 5.1, optimized interphases improve mechanical performance by balancing efficient load transfer with controlled damage evolution. Representative fracture morphologies in Figure 5 further illustrate the transition from interfacial debonding toward crack deflection, crack bridging, and improved fiber–matrix bonding, resulting in enhanced interlaminar fracture toughness and damage tolerance [24,30,37,73,74]. The mechanical benefits of interphase engineering are not defined by a single optimum structure because each loading mode requires a different balance between stiffness, compliance, and energy dissipation at the fiber–matrix interface. Therefore, interphase architectures should be tailored to the dominant loading condition rather than optimized for a single universal mechanical property.

### 5.1. Impact Resistance and Interlaminar Shear Strength

One of the primary objectives of interphase engineering is to achieve an optimal balance between efficient load transfer and damage tolerance. For glycidyl-POSS-modified aramid fibers, an interfacial layer thickness of approximately 30–40 nm at 5 wt.% POSS was reported as an effective design range, enhancing interfacial adhesion while avoiding excessive interphase brittleness [12].

Conventionally, improvements in impact resistance have relied on increasing the intrinsic toughness of the matrix, which often accompanies the well-recognized strength–toughness trade-off. Recently, Lubineau et al. proposed that this limitation can be overcome by promoting extrinsic energy dissipation during crack propagation, including crack bridging, fiber pull-out, and crack deflection, rather than simply modifying the intrinsic properties of the constituent materials. Within this framework, engineered interphases should simultaneously ensure efficient stress transfer during elastic loading while activating controlled damage mechanisms during fracture, thereby enhancing damage tolerance without compromising structural integrity. This perspective shifts the objective of interphase engineering from maximizing interfacial adhesion to controlling damage evolution through interphase architecture [75].

Consistent with this concept, CNT- and hybrid nanofiller-engineered aramid/epoxy composites have demonstrated that concurrent improvements in ILSS and impact resistance can be achieved through optimized interphase architectures. In Kevlar/epoxy composites reinforced with polyacrylonitrile/CNT/reduced graphene oxide (rGO)-modified chopped fibers, the tensile strength and modulus increased by 15.02% and 17.45%, respectively, while the ILSS increased by 67%. More importantly, the optimized interphase exhibited the lowest penetration depth, reduced strike-face cracking, and no observable interlaminar delamination following low-velocity impact, indicating that the engineered interphase effectively suppressed damage propagation through enhanced interfacial stress transfer and extrinsic energy-dissipation mechanisms [36,76]. Collectively, these findings suggest that the role of interphase engineering extends beyond maximizing interfacial adhesion alone; instead, optimized interphases should be designed to simultaneously facilitate efficient load transfer and controlled damage evolution, thereby overcoming the conventional strength–toughness trade-off while improving both structural integrity and impact tolerance.

### 5.2. Tribological Behavior

Tribological performance is strongly governed by interfacial stability. When the aramid/epoxy interface is weak, the sliding damage is readily accommodated by debonding, local fiber exposure, and unstable wear. By contrast, better-bonded Kevlar-containing epoxy systems exhibit more stable friction and wear responses, although environmental aging remains an important degradation factor. This suggests that tribological improvement should be interpreted not only in terms of hardness or surface roughness but also in terms of how effectively the interphase maintains load sharing during repeated sliding contact [55,77,78,79,80].

Environmental durability is another critical factor governing long-term interphase performance. Argon-plasma-treated aramid fiber III/epoxy composites maintained stable interfacial strength for up to 30 days of aging, with only minor changes in surface energy and roughness. Under hygrothermal conditions, molecular simulations showed that initial moisture ingress occurred preferentially through the matrix–water surface rather than by rapid wicking along the fiber–matrix interface, whereas experimental observations in Twaron/epoxy composites revealed faster moisture diffusion in the aramid fiber than in the epoxy matrix and moisture-induced debonding of surface-treated fibers. These findings indicate that engineered interphases should be evaluated under prolonged moisture exposure, UV irradiation, and cyclic fatigue, rather than only under short-term dry conditions [81,82,83].

### 5.3. DMA and Viscoelastic Interpretation

Dynamic mechanical analysis (DMA) provides valuable insights into the viscoelastic behavior of the fiber/interphase region and serves as an indirect probe of interphase quality. Comparative DMA studies of carbon- and aramid-fiber-reinforced epoxy composites have consistently shown that the storage modulus, damping behavior (tan δ), and temperature-dependent relaxation are highly sensitive to the fiber architecture and the degree of fiber–matrix constraint [59,77,84,85].

**Figure 6:**
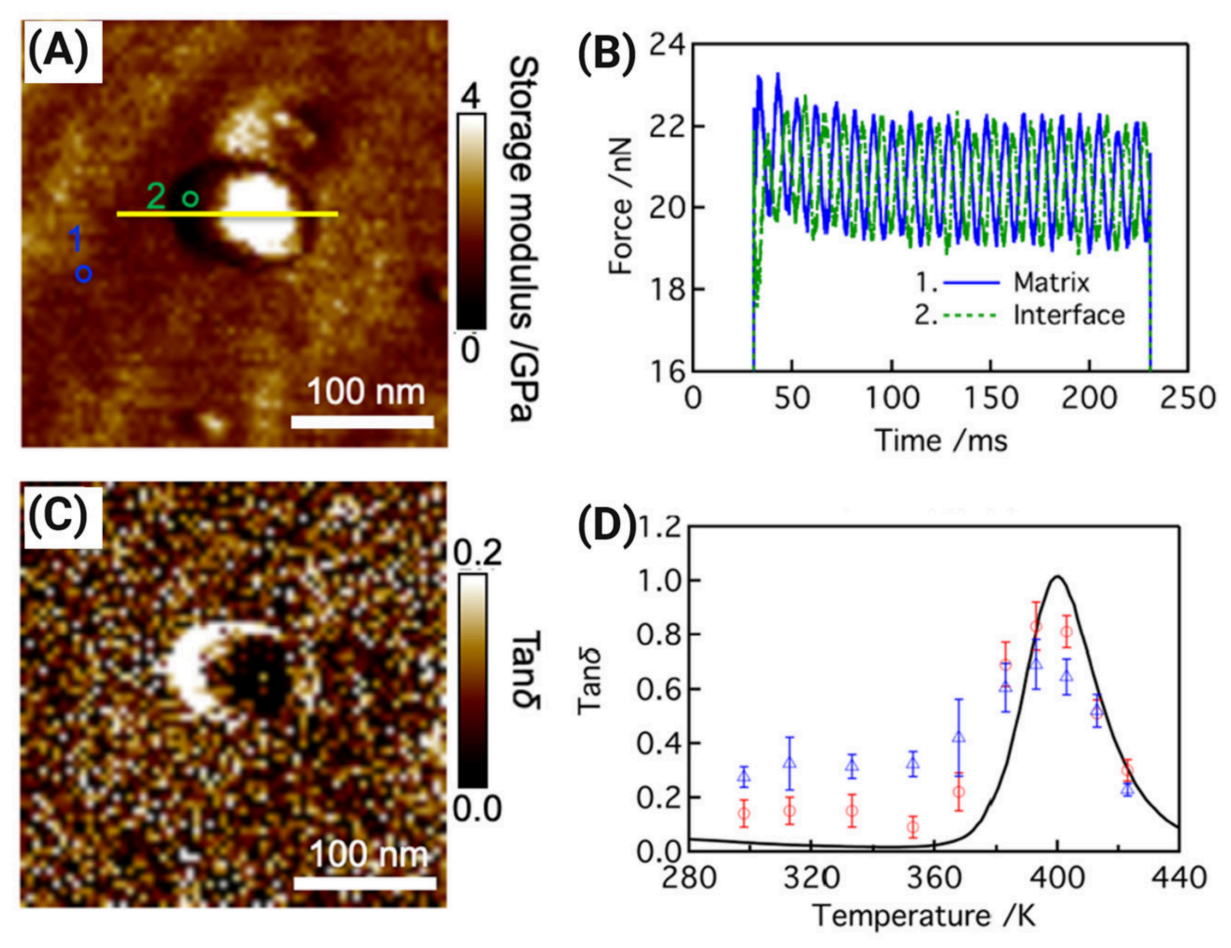
Nanoscale dynamic mechanical analysis (nDMA) of the polymer interphase. (**A**) Storage modulus map identifying the mechanically distinct interphase. The blue and green circles labeled 1 and 2 indicate the matrix and interfacial regions, respectively, from which the force oscillation curves in (**B**) were extracted. (**B**) Oscillatory force response of the matrix and interphase. (**C**) Tan δ map showing enhanced viscoelastic relaxation at the interphase. (**D**) Temperature-dependent tan δ responses of the matrix and interphase, indicating modified segmental dynamics within the constrained polymer layer. Adapted from Ref. [86].

Although conventional DMA provides only bulk-averaged responses, recent nanoscale dynamic mechanical analysis (nDMA) has directly visualized the mechanical dynamics of the polymer interphase (Figure 6). As shown in Figure 6, the interphase exhibits a mechanically distinct storage modulus (Figure 6A), enhanced viscoelastic relaxation (Figure 6C), and temperature-dependent segmental dynamics that differ from those of the bulk epoxy matrix (Figure 6D). These observations demonstrate that the macroscopic DMA response originates from altered polymer dynamics within the nanoscale interphase rather than solely from changes in bulk matrix properties.

Specifically, the interfacial polymer layer exhibits relaxation behavior distinct from that of the bulk epoxy owing to constrained segmental motion, resulting in enhanced viscoelastic energy dissipation below the glass transition temperature and slower polymer relaxation in the rubbery state. These interfacial dynamics directly influence the measured storage modulus and damping behavior and provide a mechanistic explanation for the reinforcement observed in polymer nanocomposites [86].

Accordingly, for CNT- or hybrid-nanofiller-modified aramid/epoxy composites, an increase in storage modulus accompanied by a restrained tan δ response should not simply be interpreted as stronger interfacial adhesion. Rather, it reflects constrained polymer-chain dynamics and modified segmental relaxation within the engineered interphase, which promote efficient load transfer while maintaining localized viscoelastic energy dissipation during deformation. This interpretation provides a molecular-level explanation for the enhanced mechanical performance achieved through interphase engineering.

### 5.4. Ballistic Performance

The same structure–property logic extends to ballistic loading. Kevlar/epoxy composite targets synergistically reinforced with multiwalled-CNT/graphene nanofillers exhibited a reduced projectile exit velocity and greater absorbed impact energy, showing that hybrid nanofillers can promote more efficient energy dissipation during penetration [37,87]. At the laminate scale, recent aramid armor studies have further indicated that the ballistic efficiency remains strongly dependent on the panel architecture and fabric consolidation, indicating that nanofiller-enabled interphase strengthening is particularly effective when combined with an optimized lay-up design rather than when used in isolation [88,89].

### 5.5. Summary

**Figure 7:**
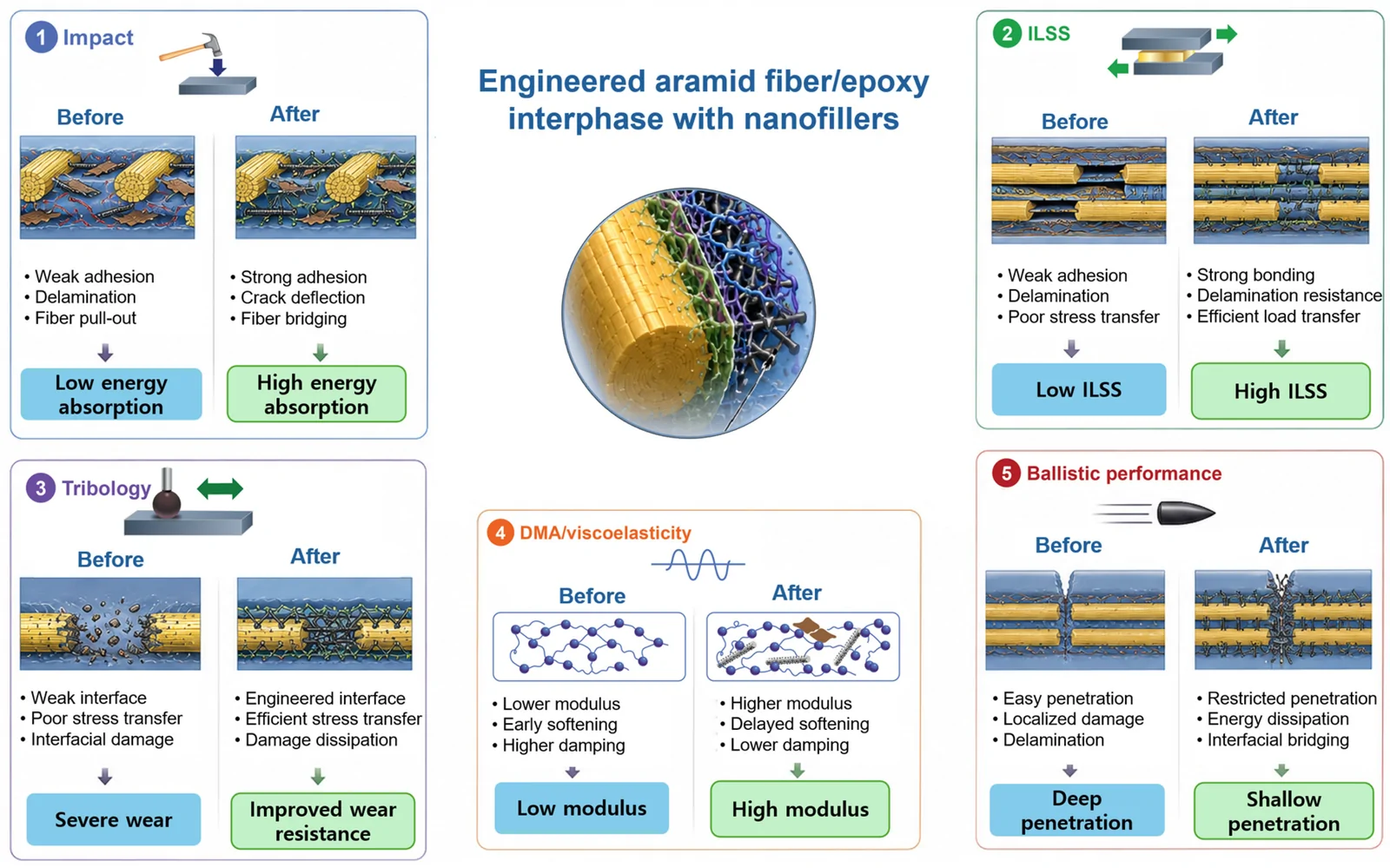
Mechanical consequences of CNT- and hybrid-nanofiller-enabled interphase engineering in aramid fiber/epoxy composites.

Overall, although the governing deformation and failure mechanisms differ depending on the loading condition, several studies have shown that interphase engineering strongly influences the interlaminar integrity, damage evolution, viscoelastic response, and energy-dissipation behavior across multiple loading environments. In this context, CNTs and hybrid nanofillers can become particularly effective, not only by stiffening the epoxy matrix but also by modifying the local interphase architecture surrounding the aramid fibers. Depending on the interphase structure and loading mode, these engineered interphases can improve stress redistribution, delay crack propagation, stabilize tribological behavior, and enhance the resistance to impact-induced or interlaminar damage. Figure 7 summarizes the representative structure–mechanism–performance relationships linking engineered interphases to various mechanical responses under different loading conditions. Rather than providing a universally optimized interface for all properties, interphase engineering enables the tailoring of the local load-transfer capability, deformability, and damage-dissipation behavior according to the dominant failure mode.

Table 4 summarizes the quantitative performance improvements of interphase- and nanofiller-engineered aramid composites under impact, tribological, dynamic mechanical, and ballistic loading conditions. The comparison demonstrates that the dominant benefits and failure-control mechanisms vary with the loading mode, highlighting the importance of tailoring the interphase architecture to the intended mechanical environment.

## 6. Future Perspectives

Future advances in aramid-fiber-reinforced epoxy composites will likely depend on moving beyond isolated surface treatments toward deliberately engineered interphases with controlled chemistry, morphology, and mechanical compliance. While many current strategies effectively enhance interfacial shear strength, future research should place greater emphasis on balancing adhesion with damage tolerance, since excessively rigid or thick interphases may suppress beneficial energy-dissipation mechanisms and lead to brittle failure under impact or complex loading. In this context, a key priority will be establishing quantitative structure–property relationships that correlate coating chemistry, interphase thickness, nanofiller localization, and cure-mediated network formation with local failure behavior and bulk mechanical performance.

At the same time, more attention should be given to scalable and non-destructive processing routes, including bio-inspired coatings, plasma-assisted reconstruction, and solvent-efficient modification platforms, with particular focus on reproducibility, coating uniformity, and compatibility with composite manufacturing. Future interphase designs may also evolve toward multifunctional architectures that combine efficient stress transfer with sensing, dielectric, thermal, or barrier functions. However, these opportunities must be validated under realistic service conditions, including moisture, thermal cycling, and long-term aging, to ensure that interfacial gains are durable and practically relevant for advanced structural applications.

Another important research direction is the integration of multiscale computational modeling with interphase engineering to establish predictive design frameworks. Molecular dynamics (MD) simulations can provide atomistic insights into interfacial bonding, polymer-chain confinement, crosslink-network evolution, and nanoscale stress-transfer mechanisms that are difficult to access experimentally. At larger length scales, finite element analysis (FEA) can quantitatively predict interphase stress distribution, crack initiation and propagation, interfacial debonding, and energy-dissipation behavior under quasi-static, fatigue, impact, and ballistic loading conditions. The integration of advanced experimental characterization with multiscale simulations will enable quantitative structure–property relationships linking interphase chemistry, morphology, and mechanical compliance to macroscopic composite performance. Furthermore, coupling these computational approaches with data-driven materials design and machine learning may accelerate the rational optimization of engineered interphases, thereby reducing reliance on empirical trial-and-error development and facilitating the design of next-generation multifunctional aramid-fiber-reinforced epoxy composites.

## 7. Conclusions

In summary, the performance of aramid-fiber-reinforced epoxy composites is governed not only by fiber or matrix properties, but more critically by how the interphase is designed. The studies reviewed here show a clear shift from conventional surface activation toward more deliberate interphase engineering strategies that regulate adhesion, stress transfer, and failure pathways. Although substantial improvements in mechanical performance have been achieved through bio-inspired coatings, hierarchical assemblies, and nanofiller-assisted architectures, the most effective designs are those that balance interfacial strength with damage tolerance rather than maximizing bonding alone. Ultimately, the evolution from conventional surface modification toward programmable interphase engineering provides a unifying framework for developing stronger, tougher, and multifunctional aramid-fiber-reinforced composites tailored for next-generation structural and protective applications.

## Figures and Tables

**Table 1:** Comparison of previous reviews and the distinctive focus of the present review.

Previous Reviews	Coverage	Primary Focus
**Palola et al. (2020)** [1]	Surface modification strategies for aramid fibers	Coating, grafting, and growth techniques to improve fiber–matrix adhesion
**Gore & Kandasubramanian (2018)** [31]	Functionalized aramid fibers and their composite applications	Surface functionalization for multifunctional and protective composites
**Zhang et al. (2021)** [32]	Surface and interface modification of aramid fiber-reinforced polymers	Physical and chemical modification for enhanced interfacial adhesion and mechanical performance
**Present Review**	Hierarchical interphase engineering in aramid fiber-reinforced epoxy composites	Interphase chemistry, hierarchical architecture, damage evolution, and structure–property–performance correlations

**Table 2:** Representative interphase-engineering strategies for aramid fiber/epoxy composites and their corresponding mechanisms, mechanical improvements, and tradeoffs.

Strategy	Interphase Formation Mode	Dominant Mechanism	Quantitative Improvement	Key Tradeoff/ Limitation	Refs.
One-step reactive surface activation (silane grafting)	Thin chemical layer (~tens of nm)	Polar group introduction, epoxy-reactive coupling, wettability improvement	IFSS increased by 50% [5] IFSS increased by 51% [6] IFSS increased by 73% [33]	Remains closer to conventional surface activation than true 3D interphase engineering	Liu et al. (2010) [5] Jia et al. (2020) [6] Li et al. (2022) [33]
Layer-by-layer (LbL) self-assembly	Programmable multilayer interphase (~hundreds of nm)	Roughness increase, charge/functional group density control, silane grafting	IFSS increased by 26% [8] ILSS increased by 43% [8]	Process sensitivity to pH, concentration, layer number; reproducibility challenges	Li et al. (2018) [8]
Bioinspired PDA + silane hybrid	PDA as universal adhesive primer + reactive silane coupling	Catechol/amine-based adhesion + subsequent silane bonding	Single-fiber pull-out increased by 63% [9]	Separating individual contributions of PDA and silane requires careful interpretation.	Sa et al. (2014) [9]
PDA/PEI bridge-layer interphase	Amine-rich bridge layer + nanofiller anchoring platform	Bridge-layer formation, filler immobilization, roughness increase	ILSS increased by 75% [11] IFSS increased by 81% [34] ILSS increased by 50.53% [4] Impact strength increased by 77.32% [4]	Excessive layer thickness risks brittle interphase and stress concentration; nonuniform filler dispersion can cause aggregation	Peng et al. (2025) [4] Xu et al. (2022) [11] Chen et al. (2026) [34]
Glycidyl-POSS grafted hybrid interphase	Nanocage structure graft + dense grafted layer (30–40 nm at 5 wt.%)	Surface polarity increase, roughness increase, epoxy-reactive glycidyl group	IFSS increased by 63% [12]	Excessive layer thickness increases brittleness and stress concentration; optimal loading exists (5 wt.%)	Li et al. (2024) [12]
Aramid nanofiber (ANF) coating	Chemically homologous ANF coating layer	Chemical affinity, roughness increase, enlarged bonding area	IFSS increased by 49.5% [7] Impact strength increased by 27.6% [7]	Coating uniformity and process reproducibility need further investigation.	Song et al. (2023) [7]
Plasma-enabled surface reconstruction	Dry-energy-based surface reconstruction	Surface aggregation state reconstruction, energy increase, active site introduction	Interfacial bonding strength increased by 66% [13]	Reads more as surface activation than precise 3D interphase design; scalability advantage strong	Wu et al. (2026) [13] Chu et al. (2014) [35]
Ionic liquid compatibilization	Molecular-level compatibilization	Compatibilization, wetting improvement, interfacial interaction tuning	Tensile strength and short beam strength increased by 14% and 17%, respectively [14]	Long-term environmental stability and residual ion effects require further evaluation.	Moraes et al. (2020) [14] Kerche et al. (2021) [15]
CNT interphase-localized design	CNT localized near fiber/matrix interphase (not bulk matrix dispersion)	Crack bridging, load redistribution, mechanical interlocking, wear stabilization	Flexural strength enhanced by 50% [27] Tensile strength, modulus, hardness, and thermal conductivity increased by 23%, 23%, 27%, and 37% [30]	Effectiveness highly sensitive to CNT dispersion and immobilization method; different behavior in epoxy vs. phenolic systems	Wang et al. (2023) [27] Singh & Dodla (2024) [30]
Hybrid nanofiller synergy (1D + 2D)	1D (CNT) + 2D (graphene oxide (GO)/reduced graphene oxide (rGO)) multiscale reinforcement	Crack bridging (1D), barrier effect and reactive sites (2D), synergistic reinforcement	Fracture toughness enhanced by 31% [24] Tensile strength, modulus, and ILSS increased by 15.02%, 17.45%, and 67%, respectively [36]	Filler content and dispersion optimization critical; excessive loading creates stress concentration sites	Kostagiannakopoulou et al. (2017) [24] Sukanya & Sundaram (2024) [36]
Multi-walled carbon nanotube (MWCNT)/ Graphene ballistic-optimized	Nanofiller synergy for energy absorption optimization	Synergistic energy absorption, exit velocity reduction	Energy absorption increased by 27.57% [37]	Performance depends on filler placement and laminate architecture; interphase design alone does not determine ballistic performance.	Nitin & Suresh Kumar (2022) [37]

**Table 3:** Comparison of aramid fiber modification methods for large-scale engineering production.

Modification	Solvent Consumption	Processing Cycle	Strength Loss	Scale-Up	Refs.
**PDA Coating**	Medium	24 h	Minimal	Low	Sa et al. (2014) [9] Xu et al. (2022) [10] Xu et al. (2022) [11] Lee et al. (2007) [16] Wei et al. (2010) [17] Jiang et al. (2011) [18]
**Plasma (DBD)**	None	Seconds/Minutes	Low	Excellent (on-line)	Xi et al. (2008) [51] Gleissner et al. (2022) [53]
**ANF Coating**	High	Days/Weeks	None	Low	Song et al. (2023) [7]
**Ionic Liquid**	Low	Minutes	None	High	Moraes et al. (2020) [14] Kerche et al. (2021) [15] Zielinski et al. (2023) [45]
**scCO_2_ Method**	None	1–4 h	None	Low (batch only)	Patadiya et al. (2023) [3] Li et al. (2019) [44] Zhang et al. (2019) [54] Zhang et al. (2020) [55]

**Table 4:** Representative quantitative improvements and dominant mechanisms of interphase- and nanofiller-engineered aramid composites under different loading conditions.

Loading Modes	Composite System	Key Metric	Improvement	Mechanism	Refs.
Impact loading	ANF, nanoparticle, and CNT/rGO-modified aramid composites	Impact strength, absorbed impact energy, tensile properties, and ILSS	Impact strength increased by 27.6% [7], Tensile strength increased by 62.0% [7], ILSS increased by 67% [36]	Improved interfacial adhesion and curing; chemically homologous interphase formation; crack bridging; nanofiller-assisted matrix toughening; enhanced load sharing and delamination suppression	Song et al. (2023) [7] Mathusoothanaperumal Sukanya and Sundaram (2024) [36]
Tribological loading	CNT-coated and hybrid aramid/epoxy composites	Specific wear rate, coefficient of friction, tensile modulus, and hardness	Specific wear rate decreased by 32.41% [30], Tensile modulus increased by 22.9% [30], Average coefficient of friction decreased by approximately 35% [79]	Increased mechanical interlocking and load sharing; reduced fiber exposure; moisture-mediated low-shear tribofilm formation; lubricating contribution of carbon fibers	Singh et al. (2024) [30] Larsen et al. (2007) [79]
Dynamic mechanical loading (DMA)	Hybrid-fiber/MWCNT composites and model epoxy interphases	Storage modulus, tan δ, and nanoscale interphase modulus	Sandwich-composite tan δ was 50% higher than that of the carbon composite [84], Storage modulus increased at 0.16 and 0.32 wt.% COOH–MWCNT loadings [85], Interphase storage modulus was 0.45 ± 0.05 GPa at 298 K [86]	Restricted polymer-chain mobility; enhanced interfacial constraint; temperature-dependent segmental relaxation; mechanically distinct nanoscale interphase behavior	Srivatsava and Sreekanth (2020) [84] Bassyouni et al. (2018) [85] Nguyen et al. (2023) [86]
Ballistic loading	Nanoparticle- and CNT/graphene-modified aramid laminates	Specific energy absorption, absorbed impact energy, penetration depth, residual velocity, back-face deformation, and ballistic limit	Specific energy absorption increased by 130% [66], Minimum penetration depth was 5.8 mm [37], Absorbed impact energy was 58.68 J [76], Ballistic limit was 632 m·s^−1^ [87]	Nanoparticle-induced matrix toughening; CNT crack bridging; graphene-assisted crack obstruction; optimized fiber orientation; multilayer load redistribution and energy dissipation	Abedi and Eslami-Farsani (2022) [66] Nitin and Suresh Kumar (2022) [37] Alagumalai et al. (2021) [76] Daungkumsawat et al. (2020) [87]

## Data Availability

No new data was created or analyzed in this study. Data sharing is not applicable to this article.
